# Ultra-Selective and Sensitive Fluorescent Chemosensor Based on Phage Display-Derived Peptide with an N-Terminal Cu(II)-Binding Motif

**DOI:** 10.3390/bios14110555

**Published:** 2024-11-14

**Authors:** Marta Sosnowska, Tomasz Łęga, Dawid Nidzworski, Marcin Olszewski, Beata Gromadzka

**Affiliations:** 1 Department of Analysis and Chemical Synthesis, Institute of Biotechnology and Molecular Medicine, Kampinoska 25, 80-180 Gdansk, Poland; m.sosnowska@ibmm.pl; 2 Nano Expo Sp z.o.o, Kładki 24, 80-822 Gdansk, Poland; 3 Department of Biotechnology, Institute of Biotechnology and Molecular Medicine, Kampinoska 25, 80-180 Gdansk, Poland; t.lega@ibmm.pl (T.Ł.); d.nidzworski@ibmm.pl (D.N.); 4 Chair of Drug and Cosmetics Biotechnology, Faculty of Chemistry, Warsaw University of Technology, Noakowskiego 3, 00-664 Warsaw, Poland; marcin.olszewski@pw.edu.pl; 5 Department of In Vitro Studies, Institute of Biotechnology and Molecular Medicine, Kampinoska 25, 80-180 Gdansk, Poland

**Keywords:** copper ion biosensor, fluorescent peptide-based sensors, N-terminal Xxx-His motif, ultra-selective Cu^2+^ chemosensor, phage display-derived Cu(II)-Xxx-His complex

## Abstract

Copper, along with gold, was among the first metals that humans employed. Thus, the copper pollution of the world’s water resources is escalating, posing a significant threat to human health and aquatic ecosystems. It is crucial to develop detection technology that is both low-cost and feasible, as well as ultra-selective and sensitive. This study explored the use of the NH_2_-Xxx-His motif-derived peptide from phage display technology for ultra-selective Cu^2+^ detection. Various Cu-binding M13 phage clones were isolated, and their affinity and cross-reactivity for different metal ions were determined. A detailed analysis of the amino acid sequence of the unique Cu-binding peptides was employed. For the development of an optical chemosensor, a peptide with an NH_2_-Xxx-His motif was selected. The dansyl group was incorporated during solid-phase peptide synthesis, and fluorescence detection assays were employed. The efficacy of the Cu^2+^-binding peptide was verified through spectroscopic measurements. In summary, we developed a highly selective and sensitive fluorescent chemosensor for Cu^2+^ detection based on a peptide sequence from a phage display library that carries the N-terminal Xxx-His motif.

## 1. Introduction

Copper is one of the harmful heavy metals among various pollutants. It is discharged into wastewater streams on a daily basis from a variety of industries, including electroplating, paints and dyes, petroleum refining, fertilizers, mining and metallurgy, explosives, pesticides, and steel. Epidemiological studies have identified a correlation between copper mining activities and a variety of health conditions, including cancer, cirrhosis, kidney failure, and headaches, in individuals residing in close proximity to copper-mining areas [1]. Copper is a critical trace element that is essential for the proper functioning of numerous enzymes, including cytochrome c oxidase, superoxide dismutase, and tyrosinase. Critical processes such as energy production, antioxidant defense, and melanin synthesis are facilitated by these enzymes. Despite its necessity, maintaining copper homeostasis is crucial. Under normal conditions, Cu^2+^ levels are tightly regulated through mechanisms that balance absorption, storage, and excretion. The liver plays a key role in distributing copper to various tissues, ensuring that other organs receive the necessary amounts for their functions. In addition, the liver regulates copper excretion primarily through bile. Excess copper is secreted into bile, which is then excreted from the body via the digestive system. This regulation ensures that copper deficiency or toxicity is minimized. An overabundance of Cu^2^⁺ ions can result in toxicity and contribute to the development of a variety of diseases. The toxicity of copper arises from its ability to engage in redox reactions, leading to the generation of reactive oxygen species (ROS) [2]. These ROS have the potential to induce oxidative stress, which can result in the destruction of cellular components, including DNA, proteins, and lipids. This oxidative damage is linked to a variety of pathological conditions, such as cardiovascular diseases, liver disorders such as Wilson’s disease, and neurodegenerative diseases like Alzheimer’s and Parkinson’s [3,4,5]. It is essential to provide a comprehensive overview of remediation techniques for various contamination scenarios, as the concentration of copper in wastewater has been reported to range from approximately 2.5 mg/L to 10,000 mg/L. Consequently, it is crucial to create wastewater removal technologies that are ecofriendly, sustainable, and relatively inexpensive. Recent years have seen a significant amount of research on a variety of methods for the removal of heavy metals from wastewater. It is advisable to establish methods for the selective and sensitive detection of Cu^2+^ ions in aqueous media and living cells, as the entire food chain is harmed by the excess of Cu^2+^ ions, and the significance of Cu^2+^ in biological activity is great.

Numerous detection methods for the sensing of copper ions have been established to date. Inductively coupled plasma, atomic absorption spectroscopy, and various electrochemical instruments have been employed. Nonetheless, they require sample pretreatment and a costly apparatus, and the samples may be compromised during the analytical procedure [6]. Furthermore, the methods are inapplicable to living organisms for bioimaging purposes. Conversely, chemosensors have garnered significant attention due to their potential to address these deficiencies. They are user-friendly, economical, and responsive. Chemosensors utilizing fluorescence can be employed for the real-time bioimaging of diverse analytes.

Fluorescence detection assays represent one of the most straightforward and convenient methodologies among various detection techniques, offering simplicity, sensitivity, and versatility for the identification of a broad spectrum of analytes. Recent advancements in nanoscale science and engineering have produced nanomaterials exhibiting distinctive optical properties. In the last ten years, various fluorescent nanoprobes have been created for the highly sensitive and selective detection and imaging of metal ions, both in vitro and in vivo [7,8]. Organic dyes, including rhodamine, fluorescein, and cyanine, are the predominant fluorescent probes; however, their limitations in terms of photostability, pH sensitivity, and potential toxicity make them less ideal for long-term applications in biological imaging. The aforementioned limitations have prompted the creation of alternative fluorescent probes for metal ions characterized by enhanced luminescence, superior photostability, and improved biocompatibility, facilitated by advancements in nanoscale science and engineering. The dansyl fluorophore is extensively utilized in the modification of amino acids, protein sequencing, and amino analysis due to its elevated fluorescence quantum yields and substantial Stokes shift [9,10].

Peptide-based fluorescent chemosensors for metal ion detection have many advantages compared with other small organic molecules [11,12]. The remarkable specificity and sensitivity of biodegradable peptides as ligands for detecting heavy metals in aqueous systems have garnered significant attention. Peptides contain a range of potential donor atoms, and the complexes formed exist in a variety of conformations. Therefore, short sequences of amino acids can be engineered to take advantage of peptides’ high affinity for binding particular target molecules like heavy metal ions. This significantly improves their potential for the development of sensors, particularly in the fields of environmental monitoring and water quality assessment. One method that makes the ability to precisely identify unique amino acid sequences (peptides) for the detection of heavy metal ions is phage display technology. The phage display requires the use of phage libraries, which consist of diverse phages displaying distinct peptides or proteins on their surfaces. Phages with a strong affinity for a compound can be isolated via an affinity selection process. Determining the peptides presented on these specific phages can be accomplished by sequencing the gene that encodes the peptide. The efficacy of the phage display is beneficial for the toxicological evaluation of chemical substances by utilizing it as a screening mechanism for identifying the most important targets. Phage display selection technology has been employed to identify specific peptides that selectively bind to cancer cells, proteins, nanoparticles, and metal ions, including Cd(II), Ni(II), Co(II), Pb(II), Cu(II), and Cr(III), since its initial development [13,14,15,16,17,18].

It is widely known that peptides and proteins with N-terminal motifs such as NH_2_-Xxx-His (XH) and NH_2_-Xxx-Zzz-His (XZH) form well-established Cu(II) complexes [19,20,21,22]. Thus, these motifs have been utilized to obtain peptides with specific functions. One of the most straightforward methods for adding a high-affinity Cu(II) binding site to virtually any peptide or protein is to incorporate XH or XZH into the structure through chemical or recombinant methods [23].

The objective of this research was to identify specific amino acid sequences through phage display technology that can be employed in the development of an ultra-selective and sensitive fluorescent chemosensor for Cu^2+^ detection. The results indicate that the identified peptide sequence containing the N-terminal Xxx-His motif exhibited a high selectivity toward Cu^2+^ ions. Moreover, when the selected sequence (P-11) was modified with the dansyl fluorophore at the N-terminus and KW amino acids at the C-terminus, the ability to detect copper(II) ions with a high sensitivity and selectivity remained intact.

## 2. Materials and Methods

### 2.1. Materials

Metal salt solutions at 20, 200 µM and 10 mM of cadmium chloride, copper(II) chloride, nickel(II) chloride, mercury(II) chloride, and manganese(II) chloride were prepared with deionized water; 0.05% TBST buffer (50 mM Tris-HCl, 150 mM NaCl, 0.05% TWEEN 20 (*v*/*v*) pH 7.4); glycine buffer for elution was composed of 0.2 M glycine-HCl, pH 2.2. 10 mM Nα,Nα-Bis(carboxymethyl)-L-lysine hydrate (BCML) in deionized water (Sigma Aldrich, St. Louis, MO, USA); and maleic anhydride activated plates (Thermo Scientific Chemicals, Waltham, MA, USA) were used.

Fmoc–Rink amide AM resin (0.7 mmol/g) was acquired from Iris Biotech GmbH (Marktredwitz, Germany). Fmoc protected amino acids Fmoc-Met-OH, Fmoc-Ile-OH, Fmoc-Val-OH, Fmoc-Pro-OH, Fmoc-Glu(OtBu)-OH, Fmoc-Lys(Boc)-OH, and Fmoc-Trp(Boc)-OH were purchased from CSBio Ltd. (Shanghai, China). Fmoc-His(Boc)-OH was from CEM Corporation (Matthews, NC, USA). 5-Dimethylaminonaphthalene-1-sulfonyl chloride (DNS), Oxyma pure, N,N′-diisopropylcarbodiimide (DIC), triethylamine (TEA), piperidine, 99%, extra pure, triisopropylsilane (TIS), 4-(2-hydroxyethyl)piperazine-1-ethanesulfonic acid (HEPES), Pb(NO_3_)_2_, and MnCl_2_ were obtained from Sigma-Aldrich (St. Louis, MO, USA). N,N-Dimethylformamide (DMF) (99.8%) and trifluoroacetic acid (TFA) for synthesis, chromium standard solution, cadmium standard solution, Hg(NO_3_)_2_, CuSO_4_, ZnSO_4_, and NiCl_2_ were employed. NaAsO_2_, NaCl, and KCl were acquired from VWR International, LLC (Radnor, PA, USA). Ethyl dieter was collected from POCH S.A (Gliwice, Poland).

Acetonitrile of HPLC gradient-grade, acetonitrile hypergrade for LC-MS LiChrosolv^®^, water (LC-MS LiChrosolv^®^, Merck, St. Louis, MO, USA), trifluoracetic acid for the HPLC, and formic acid (FA) (LC-MS LiChropur™) were purchased from Sigma-Aldrich (St. Louis, MO, USA). Doubly distilled water (Hydrolab-Reference purified) with conductivity not exceeding 0.05 µS/cm was used. All other chemicals used were of analytical reagent-grade unless otherwise noted.

### 2.2. Phage Library and Bacterial Strain

The Ph.D.™-7 Phage Display Peptide Library Kit was acquired from NEB (New England Biolabs GmbH, Frankfurt am Main, Germany). The host strain E. coli K12 ER 2738 (NEB) was cultivated overnight at 37 °C in an LB medium supplemented with 20 μg/mL tetracycline (stock solution 20 mg/mL in a 1:1 water/ethanol mixture, preserved at −20 °C) and subsequently stored in 100 μL aliquots in PCR tubes at −80 °C until required. The strain was preserved throughout the ongoing experiments on LB agar plates (10 g/L tryptone, 5 g/L yeast extract, 5 g/L NaCl, 15 g/L agar with 20 μg/mL tetracycline).

### 2.3. Immobilization of Copper Ions on Plates

One hundred microliters of 10 mM Nα,Nα-Bis(carboxymethyl)-L-lysine hydrate in 0.1 M NaPO_4_, pH 8, was dispensed into each well of a maleic anhydride-activated microtiter plate and incubated overnight at ambient temperature. The plate was subsequently rinsed thrice with 300 µL of 0.05% TBST buffer. The plate was obstructed by incubation with 3% BSA in 0.05% TBST buffer for 2 h at room temperature. The plate was subsequently rinsed thrice with 300 µL of 0.05% TBST buffer. The plate was subsequently incubated with 10 mM metal salts for 20 min at room temperature. Modified plates were used for biopanning.

### 2.4. Surface Panning Procedure

This study utilized the host bacterial strain *E. coli* K12 ER 2738 (NEB) and the Ph.D.™-7 Phage Display Peptide Library Kit. The culture was incubated overnight at 37 °C in an LB medium supplemented with 20 μg/L tetracycline and preserved in 100 μL aliquots in PCR tubes at −80 °C for future use. The strain was preserved on LB agar plates during the experiments. The plates contained 10 g/L tryptone, 5 g/L yeast extract, 5 g/L NaCl, 15 g/L agar, and 20 μg/L tetracycline. The phage display library was screened for metallospecific peptides following the manufacturer’s instructions, with minimal modifications. A negative selection step was integrated into the protocol to remove nonspecific phage clones from the library bound to metal ions other than copper. In summary, 10^11^ PFU of the library was diluted in 100 μL of 0.05% TBST buffer and subsequently introduced to a plate coated with a 10 mM nontarget metal ion. Following a 60 min incubation at room temperature with a plate shaker set to 100 rpm, unbound phage clones were transferred into wells coated with an additional negative target and incubated for another 60 min at room temperature with the plate shaker at 100 rpm. All clones underwent the negative selection process. Negative selection was conducted against nickel, manganese, cadmium, and mercury. Subsequent to negative selection, the manufacturer’s guidelines were adhered to in order to perform three rounds of biopanning with the designated metal ion. Two hundred and forty plaques were chosen from IPTG/X-gal LB titration plates after the third round to amplify phage clones for DNA sequencing. Subsequently, the 7-amino acid copper ion-binding sequences were extracted through the sequencing of individual phages.

### 2.5. Binding Specificity Assay

First, 100 µL of 10 mM Nα,Nα-Bis(carboxymethyl)-L-lysine hydrate in 0.1 M NaPO_4_, pH 8, was dispensed into each well of a maleic anhydride-activated microtiter plate and incubated overnight at room temperature. The plate was subsequently rinsed thrice with 300 µL of 0.05% TBST buffer. The plate was blocked by incubating with 3% BSA in 0.05% TBST buffer for 2 h at room temperature. The plate was subsequently rinsed thrice with 300 µL of 0.05% TBST buffer. The plate was subsequently incubated with 20 µM metal salts for 20 min at room temperature. Plates coated with metal ions were incubated with 100 µL of 10^9^ PFU of the selected clones in TBS buffer per well. The plates were incubated for 60 min at room temperature with shaking at 100 rpm on a plate shaker. Unbound phages were eliminated and the plate was rinsed ten times with 300 µL of 0.05% TBST buffer. For phage elution, 100 µL of 0.2 M glycine-HCl (pH 2.2) was utilized, followed by a 15 min incubation at room temperature, after which 15 µL of 1 M Tris-HCl (pH 9.1) was incorporated. Eluted phages were tittered following the procedure outlined in The Ph.D.™-7 Phage Display Peptide Library Kit manual utilizing LB/IPTG/Xgal plates. Binding specificity is expressed as the phage titer eluted from wells coated with the chosen metal.

### 2.6. Peptide Synthesis, Modification, and Characterization

Using microwave-assisted Fmoc solid-phase peptide synthesis (SPPS), the peptide with the sequence Dansyl-Met-His-Ile-Pro-His-Glu-Lys-Trp-NH_2_ was synthesized on the Rink amide resin (0.1 mmol). Peptide chain elongation was performed utilizing an Initiator+ Alstra™ (Biotage, Uppsala, Sweden) automated microwave peptide synthesizer. Couplings were carried out twice for 5 min at 75 °C utilizing the Fmoc amino acid (5 equiv.), DIC (5 equiv.), and Oxyma (5 equiv.) in DMF. The deprotection of the Fmoc group utilized a 20% piperidine solution in DMF at room temperature (1 × 3 min, 1 × 10 min). DNS was introduced at the N-terminal side of the peptide in a separate step. To this end, DNS (5 equiv.) and TEA (3 equiv.) were introduced to the peptidyl resin in darkness for 4 h. The DNS-labeled peptide was cleaved from the Rink amide resin utilizing a cleavage solution of TFA/TIS/H_2_O (95:2.5:2.5) for 2 h, precipitated with anhydrous, cold diethyl ether, and subsequently lyophilized. The resultant product was characterized utilizing a reverse-phase HPLC Shimadzu system (Prominence-i LC-2030C Plus, Shimadzu, Kyoto, Japan) equipped with a Jupiter 4 µm Proteo, 90Å, 4.6 × 250 mm column, employing UV detection at λ = 224 nm. A linear gradient method was applied, transitioning from 5 to 95% solvent B over 60 min at a flow rate of 1 mL/min, where solvent A was water and solvent B was acetonitrile, both containing 0.1% TFA. Electrospray ionization mass spectrometry (ESI MS) in positive ion mode was conducted utilizing a single quadrupole mass spectrometer (LCMS 2020 Shimadzu, Kyoto, Japan). Isocratic elution at 60% B, with eluent A comprising water and 0.1% formic acid (LCMS grade) and eluent B consisting of acetonitrile (LCMS grade) with 0.1% formic acid was conducted at a flow rate of 1.5 mL/min.

### 2.7. General Spectroscopy Measurements

DNS–P-11, a highly soluble fluorescent peptide, was dissolved in double-distilled water to create a 2 mM DNS–P-11 solution, which was stored at 4 °C. The solutions of metal ions were derived from Hg(NO_3_)_2_, Pb(NO_3_)_2_, MnCl_2_, ZnSO_4_, CuSO_4_, and NiCl_2_. Standard solutions of Cd, Cr, NaAsO_2_, KCl, and NaCl in double-distilled water at a concentration of 10 mM were used. Subsequent to appropriate dilution, the resultant stock solution was utilized for all spectral measurements. The Synergy H1MG Multimode Microplate Reader (BioTek Instruments, Winooski, VT, USA) was employed to assess the fluorescence spectra on a 96-well plate featuring an F-bottom (Greiner Bio-One International GmbH, Kremsmünster, Austria) with a working volume per well of 200 μL. The excitation wavelength for the dansyl fluorophore was established at 330 nm.

## 3. Results and Discussion

### 3.1. Phage Display-Derived Unique Peptide for Cu^2+^ Ion Detection

**Figure 1:**
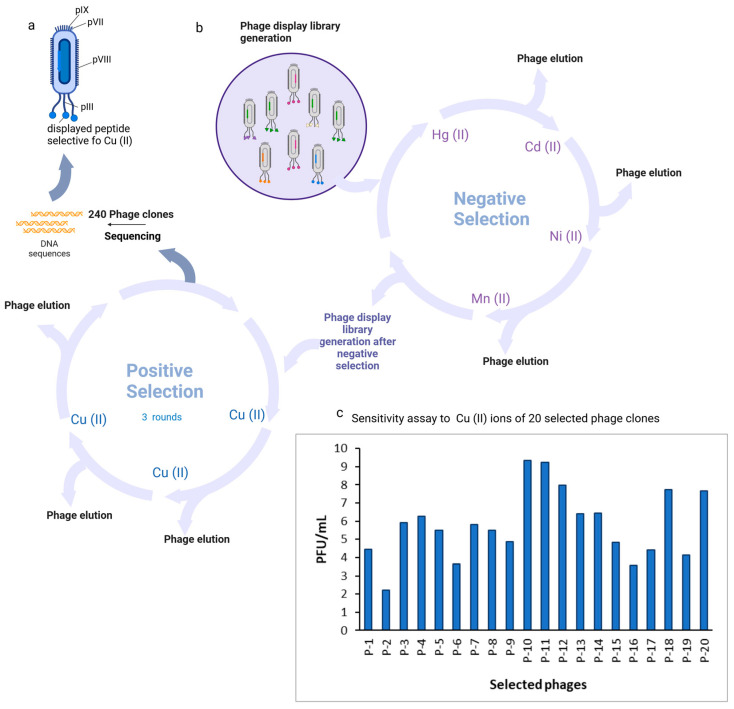
Identification of M13 phages selective for Cu(II) ions. (**a**) Schematic representation of the M13 phage clone; (**b**) schematic representation of the modified biopanning procedure. The phage library kit Ph.D.™-7 phage display peptide was used to conduct an overnight experiment in an LB medium at 37 °C with the host bacterial strain *E. coli* K12 ER 2738. The protocol was modified to include negative selection steps in order to eliminate nonspecific phage clones bound to metal ions other than copper from the library. In conclusion, 10^11^ PFU of the library was diluted in 100 μL of 0.05% TBST buffer and subsequently added to a plate that was coated with a 10 mM non-target metal ion. Unbound phage clones were pipetted into wells that were coated with an additional negative target following incubation. Nickel, manganese, cadmium, and mercury ions were subjected to negative selection. Three rounds of biopanning were conducted with the target metal ion following negative selection. Following the third round of biopanning, M13 phage plaques were selected from titration plates to amplify phage clones for DNA sequencing; (**c**) binding affinity and selectivity of phage clones. Comparison of the binding affinity to Cu(II) among the 20 selected phages represented by PFU/mL functionalized and modified with a Cu(II) ion microtiter plate were incubated with 100 µL of 10^9^ PFU selected clones per well. Non-bound phages were discarded and the plate was washed 10 times. For phage elution, 100 µL of 0.2 M glycine-HCl (pH 2.2) was used. Eluted phages were tittered according to the method described in the Ph.D.™-7 Phage Display Peptide Library Kit manual. Binding specificity is presented as the phage titer eluted from wells coated with Cu^2+^ ions. Three independent experiments were conducted and the mean is presented. Created in BioRender Premium accessed on 1 September 2024, BioRender.com/x91b707.

After the entire biopanning procedure, 240 monoclonal Cu(II)-binding M13 virus plaques containing phage monoclonal peptides were isolated for phage amplification and DNA sequencing. DNA sequences were successfully obtained for all of the clones. A total of 20 individual clones containing distinctive peptides were identified from the selected 240 plaques. The biopanning procedure proved effective, demonstrated by the increased binding affinity of phage clones with Cu(II)-binding peptides to Cu(II). P-10, P-11, P-12, P-18, and P-20 demonstrated the greatest affinity (Figure 1).

### 3.2. Characterization and Amio Acid Sequence Analysis of Unique Phage Display-Derived Peptides

The analysis of the amino acid sequences of the twenty selected phages revealed that only four of them had a histidine residue: P-2 (GVKMHTH), P-4 (NLIHKHS), P-10 (DTAHGTW) P-11 (MHIVPHE), and P-19 (HLTSPML), with P-10 and P-11 showing the greatest selectivity towards Cu^2+^ ions. Thus, P-10 and P-11 peptide sequences were taken into consideration due to the well-known, potent, and short N-terminal motif, which have a His residue in the second or third position. P-10 with a sequence DTAHGTW, featured histidine at fourth position, however, the possibility of formation of chelate system is removed if there are two or more intervening amino acid residues between the N-terminal amine the imidazole donors.

**Figure 2:**
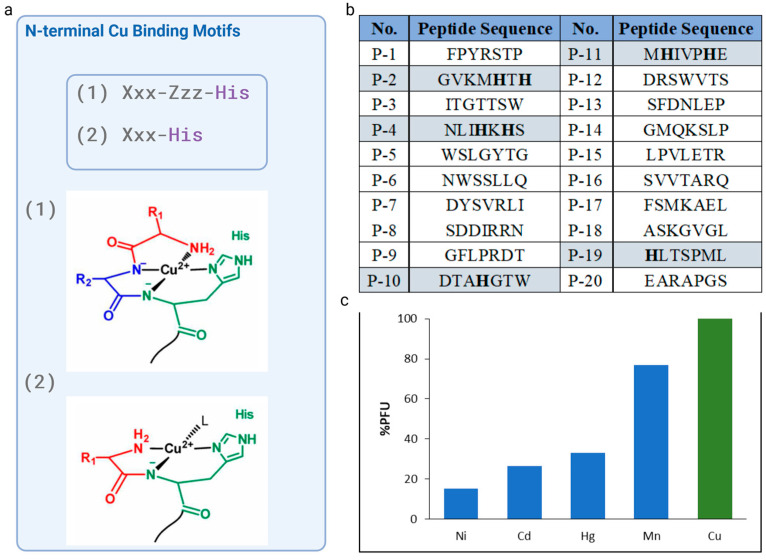
Analysis of the amino acid sequences in phage display-derived peptides: (**a**) scheme of the equatorial Cu(II) coordination of the Xxx-His and XZH motifs. L depicts an external ligand, and could be a solvent or other ligand; (**b**) list of the selected peptides with an analysis of N-terminal Cu(II)-binding motif presence; (**c**) cross-reactivity assay of M13-Cu(II)-peptide. The microtiter plate was functionalized and modified with Cu(II), Mn(II), Hg (II), Ni (II), and Pb(II) ions, and 100 µL of 10^9^ PFU selected clones were incubated per well. The plate was washed ten times, and non-bounded phages were discarded. Then, 100 µL of 0.2 M glycine-HCl (pH 2.2) was used for phage elution. Eluted phages were titrated in accordance with the procedure outlined in the manual for the Ph.D.™-7 Phage Display Peptide Library Kit. As the phage titer was eluted from wells coated with different metal ions, binding cross-reactivity and specificity was demonstrated. The mean of three independent experiments is presented. Created in BioRender Premium accessed on 1 September 2024, BioRender.com/x91b707.

Since histidine residue located in position two of the peptide chain allows for the generation of a highly desirable Cu^2+^ complex, P-11, with the sequence MHIVPHE, which possessed a His residue at the second position with respect to the free N-terminal amine and high affinity toward Cu^2+^ ions, was chosen as the candidate peptide for further examination. The NH_2-_-Xxx-His motif generated a highly stable Cu^2+^-Xxx-His complex [21,24]. Peptides contained N-terminal amino acid sequence Xxx-His, where Xxx can be any amino acid, except Pro, display a high affinity for Cu(II). In this manner, Cu^2+^ can bind to XH in a tridentate fashion, with three nitrogens from the N-terminal amine (NH_2_), the first amide (Nα), and imidazole nitrogen at the delta ring position (Nδ) being involved (Figure 2a). The fourth equatorial binding site was occupied by an external molecule such as water, buffer, etc.

Following a thorough investigation and the selection of the sequence, four supplementary metal ions (Ni, Cd, Hg, and Mn) were used in the cross-reactivity assays to enhance selectivity investigations. The selective Cu(II)-binding M13 phage clone that displaces the MHIVPHE peptide demonstrated a strong affinity for copper(II) ions while exhibiting minimal affinity for other metal ions, such as Ni, Cd, Hg, and Mn. This result is of great importance, as the selectivity of the chemosensor for specific ions is crucial.

### 3.3. Design of Modifications and Solid-Phase Peptide Synthesis

**Figure 3:**
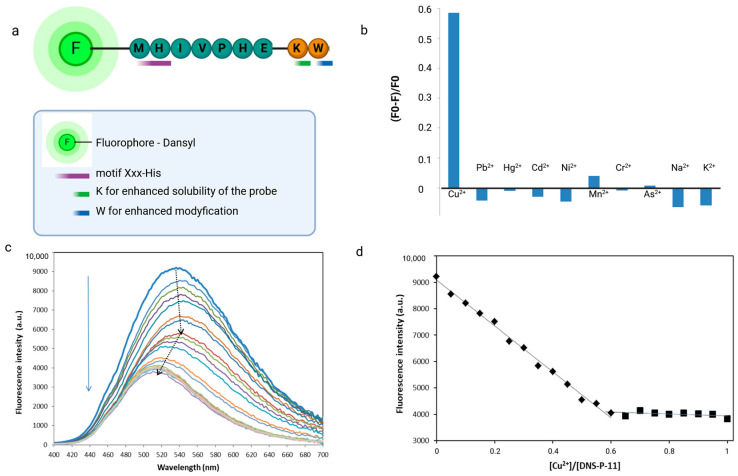
Design, modification, and characterization of fluorescent chemosensors based on phage display-derived peptide for Cu^2+^ detection. (**a**) Schematic representation of design peptide-based chemosensor. (**b**) The selectivity of the of DNS–P-11 peptide (10 µM) for the Cu^2+^ assay. The concentration of all tested ions was 10 µM (HEPES buffer 50 mM, pH 7.41). F0 and F were the fluorescence intensities of DNS–P-11 in the absence and presence of metal ions. (**c**) Fluorescence titration spectra of DNS–P-11 (10 µM) with the addition of Cu^2+^ (0–1.0 equiv.) in HEPES buffer (50 mM, pH 7.41) solutions. (**d**) The corresponding titration profile as a function of Cu^2+^ concentration. Created in BioRender. Premium accessed on 1 September 2024, BioRender.com/x91b707.

The unique selective native peptide sequence Met-His-Val-Ile-Pro-His-Glu (P-11) selected from phage display experiments was modified to obtain specific optic functions of peptide. Metal ion-binding peptide was synthesized on the Rink amide resin by Fmoc/tBu microwave-assisted solid-phase peptide synthesis [25]. The fluorophore (dansyl chloride) and tryptophan as donors were conveniently attached during SPPS, eliminating the need for labeling reactions. Dansyl (DNS) was introduced at the N-terminal side of the peptide, tryptophan residue was inserted at the C-terminus manually, in the separate step. Therefore, a Trp residue was inserted into the native peptide sequence to realize the sensing of Cu^2+^ ions by FRET mechanisms. A flexible linker-like lysine residue was inserted between native sequence and donor fluorophore to increase the peptide’s solubility, minimize steric hindrance, and ensure proper positioning of the fluorophore (Figure 3). Labeling peptides by the use of dansyl chloride (DNS-Cl) is a versatile and highly sensitive means of fluorescent detection. DNS was chosen for the construction of the chemosensor due to its strong fluorescence, relatively long emission wavelength in the visible region, and rapid reactivity with primary amines. Moreover, sequence labeling with dansyl fluprophore allows for very rapid detection at a low quantity in fluorescent spectroscopy. DNS typically exhibits strong fluorescence at 540 nm when excited at an appropriate wavelength, usually around 330 nm [9,26].

Obtained peptide was cleaved from the resin, isolated, and lyophilized. The successful synthesis was confirmed by ESI mass spectrometer. The crude peptide was purified by HPLC. Peptide DNS–P-11 characterization: yellowish solid; synthetic yield: 89%; HPLC purity > 95%; Rt 20.708 min, ESI MS of peptide wsas calculated as 1408.4 (g/mol); observed value as [M + 2H]^2+^ *m*/*z* = 706, [M + 3H]^3+^ *m*/*z* = 471.

### 3.4. Metal Ion Selectivity Experiment

The fluorescence spectroscopic response of DNS–P-11 peptide (10 µM) toward metal ions (Cu^2+^, Hg^2+^, Pb^2^⁺, Cd^2^⁺, Ni^2^⁺, Mn^2^⁺, Cr^3+^, As^3+^, Na^+^, and K^+^) was evaluated in HEPES buffer solutions (50 mM, pH 7.41). Separate solutions of each metal ion prepared at concentrations of 10 µM were individually added to the DNS–P-11 solutions and fluorescence emission spectra were recoded with excitation at 330 nm. Comparing the fluorescence response of DNS–P-11 in the presence of Cu^2+^ ions and other selected metal ions in the tested concentration, it was found that DNS–P-11 exhibited selectivity for Cu^2+^ in aqueous solution and no fluorescent response to other metal ions. Conversely, after adding 1.0 equiv. Cu^2+^ ions, the emission intensity of DNS–P-11 was significantly reduced, with the fluorescence quenching rate for Cu^2^+ of about 58.5% (Figure 3b). The fluorescence spectra in Figure 3c exhibit biphasic behavior, indicating that (most likely) depending on the concentration, Cu^2+^ binds the DNS–P-11 peptide with different binding constants.

### 3.5. Characterization of the Sensing Process of a Fluorescent Chemosensor Based on Phage Display-Derived Peptide for Cu^2+^ Detection

In order to better understand the sensing process, the fluorescence spectra changes of fluorescent peptide, DNS–P-11 with Cu^2+^ ions was demonstrated. For this purpose, the different concentrations of Cu^2+^ ion solutions (0–10 µM) were added to 10 μM DNS–P-11 in HEPES buffer solutions (50 mM, pH 7.41), and fluorescence spectra of the obtained solutions were recorded. As illustrated in Figure 3c, the fluorescence emission peak of DNS–P-11 gradually decreased with increasing Cu^2+^ concentration. The fluorescence spectroscopy of DNS–P-11 indicated that the addition of Cu^2+^ (ranging from 0–1.0 equiv.) caused a decrease in fluorescence at 540 nm.

Notably, the fluorescence intensity of DNS–P-11 at 540 nm began to gradually quench after the addition of Cu^2+^ ions (0.05–0.45 equiv.), while the emission maximum was shifted to 546 nm. An increase in Cu^2+^ concentration (0.5–1.0 equiv.) resulted in a further decrease in fluorescence intensity with a concurrent blue-shifted emission peak 36 nm from 546 to 510 nm (Figure 3c). According to the trend diagram of fluorescence titration, the fluorescence titration curve of DNS–P-11 reached a steady plateau after the addition of 0.65 equiv. of Cu^2+^, while the increasing level of Cu^2+^ up to 1 equiv. maintained the saturation of DNS–P-11-Cu^2+^ complex at constant level (Figure 3d). The above results determined the binding stoichiometry of 2:1 between DNS–P-11 and Cu^2+^ ions.

The results of the fluorescence titration experiment clearly demonstrated that DNS–P-11 exhibits a high level of selectivity toward Cu^2+^ ions by decreasing fluoresce emission (Figure 3).

**Figure 4:**
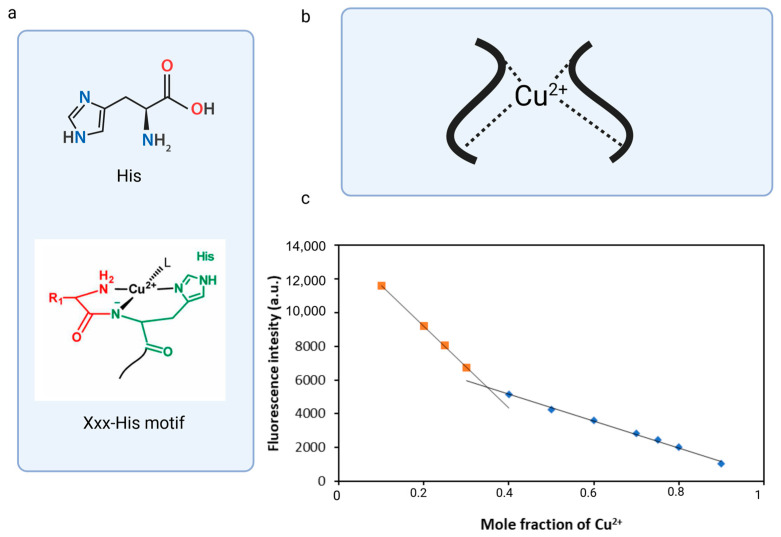
Binding stoichiometry of the DNS–P-11 peptide. (**a**) Schematic representation of the XH motif. (**b**) Schematic representation of the DNS–P-11 peptide with the Cu-binding motifs. (**c**) Job’s plot analysis curve. Created in BioRender. accessed on 1 September 2024, BioRender.com/x91b707.

Another way to determine the binding stoichiometry of fluorescent peptide and metal ions is to determine Job’s plot analysis curve. A typical Job’s plot shows a curve that reaches a maximum at the mole fraction corresponding to the stoichiometric ratio of the complex. In some cases, as the complex forms, the measured value (e.g., fluorescence intensity) decreases, causing the Job plot to exhibit a downward trend and reach a minimum of the measured value at a specific mole fraction. Such a scenario occurred during the complexation of DNS–P-11 with Cu^2+^, during which the fluorescence intensity decreased, that is inversely related to the amount of complex formed. The complex’s stoichiometry was determined using a total concentration of DNS–P-11 and Cu^2+^ at 10 μM. The result exhibited the inflection point at 0.35 mol fraction for Cu^2+^ in 50 mM HEPES buffer solution, which could be consistent with the formation of 2:1 coordination stoichiometry for DNS-P-11 to Cu^2+^ [27] (Figure 4c). The stoichiometric ratio of the complex calculated using Job’s plot was in accordance with the results of the fluorescence titration experiment (Figure 3d). The coordination capability of the peptides with the N-terminal Xxx-His motif relative to the transition metal ions could be significantly reduced by blocking the N-terminal amino group; however, it is believed that the second peptide molecule significantly influenced the metal ion binding process and stabilized the complex.

### 3.6. Analysis of the Selectivity and Sensitivity of a Fluorescent Chemosensor (DNS–P-11) Based on Phage Display-Derived Peptide for Cu^2+^ Detection

**Figure 5:**
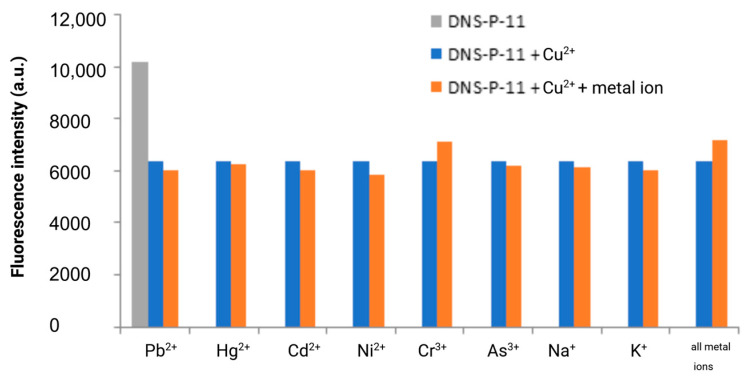
Fluorescence response of DNS–P-11 (10 µM) to Cu^2+^ (1.0 equiv.) in the presence of tested metal ions (5.0 equiv.) in HEPES buffer solutions (50 mM, pH 7.41). Created in BioRender Premium accessed on 1 September 2024, BioRender.com/x91b707.

The specificity of fluorescence-based detection methods is crucial in many applications, including clinical diagnostics and environmental monitoring. To ensure this specificity, it is essential to test the response of the DNS–P-11 peptide to its target ions in the presence of other potentially interfering metal ions. The fluorescence responses of DNS–P-11 to Cu^2+^ (1.0 equiv.), in the presence of other tested metal ions (5.0 equiv.) were measured in HEPES buffer solutions (50 mM, pH 7.41). Moreover, we also explored the ability of DNS–P-11 to detect Cu^2+^ in a mixture of all tested ions. Cross-reactivity refers to the response of the peptide DNS–P-11 to multiple metal ions, which may affect the interpretation of the fluorescence signal. Thus, to verify the peptide biosensor’s selectivity for Cu^2+^ ions, the fluorescence responses of DNS–P-11 to Cu^2+^ (1.0 equiv.), in the presence of other tested metal ions (5.0 equiv.) were measured in HEPES buffer solutions (50 mM, pH 7.41). The experimental results showed that the presence of other metal ions, such as Pb^2+^, Hg^2+^, Cd^2+^, Ni^2+^, and Mn^2+^ or trivalent chromium and arsenic ions, had no effect on the response of DNS–P-11 to Cu^2+^ and also had no impact on the interpretation of the fluorescence signal, as demonstrated in Figure 5. This clearly indicated formation of a stable complex between DNS–P-11 and Cu^2+^ ions and strongly support ability of DNS–P-11 to detect Cu^2+^ in the presence of other metal ions. In addition, the simultaneous introduction of all the analyzed ions (5 equiv. of each) into the sample containing the DNS–P-11 peptide probe and Cu^2+^ ions (1 equiv.) did not substantially impact the reduction of the fluorescence emission of the peptide–Cu^2+^ complex. This property is crucial for developing selective biosensors that accurately identify and measure Cu^2+^ in complex mixtures.

The results demonstrate that the fluorescence can be controlled by an ON–OFF switch, which is in line with the powerful chelating capability of the DNS–labeled peptide towards Cu^2+^. It is important to highlight that the DNS–P-11 peptide remains highly responsive to Cu^2+^ ions even at high concentrations of other competitive ions, which emphasizes the ability of the designed DNS–P-11 peptide for selective copper(II) ion detection in aqueous systems.

### 3.7. Detection Limit of DNS–P-11 for Cu^2+^ Ions

**Figure 6:**
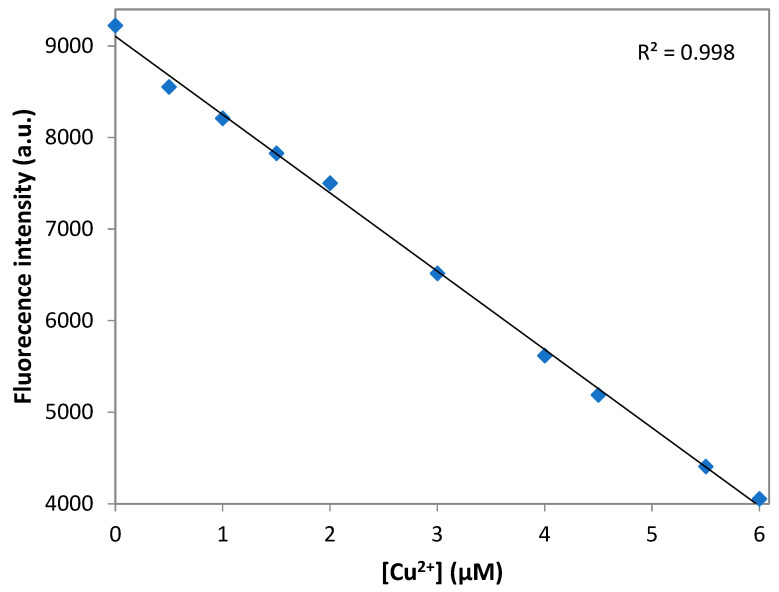
Detection limit of DNS–P-11 for Cu^2+^ ions. Linear fluorescence emission intensity of DNS–P-11 in HEPES buffer (50 mM, pH 7.41) solution as a function of Cu^2+^ concentration.

The limit of the detection (LOD) was calculated according to the fluorescence titration experiment. In to ascertain the S/N ratio, the fluorescence emission intensity of DNS–P-11 in the absence of Cu^2+^ was measure six times and standard deviation of blank measurement was obtained. Three independent duplicate measurements of fluorescence emission intensity were evaluated in the presence of Cu^2+^ ions and the mean intensity was plotted as function of Cu^2+^ for determining the slope. Then LOD for Cu^2+^ was obtained by the equation: LOD = 3σ/a, where σ is the standard deviation of the response, a is the slope of the calibration curve of the plot. LOD for the DNS–P-11 biosensor for Cu^2+^ was calculated using the linear relationships of fluorescence emission intensity at maximum wavelength with the addition of increasing concentrations of Cu^2+^. Figure 6 shows a good linear relationship between fluorescence emission intensity of DNS–P-11 and Cu^2+^ concentration solution in the range of 0–6 µM, in 50 mM HEPES buffer, with correlation coefficient R^2^ = 0.998. According to the equation of LOD = 3σ/a, the detection limit of DNS–P-11 for Cu^2+^ is 0.46 µM. The data indicate that the DNS–P-11 chemosensor is highly sensitive to Cu^2+^ ions, and the detection limit is significantly lower than the WHO-recommended limit value of 31 µM.

## 4. Conclusions

The high value of peptides in the development of chemosensors for a variety of applications, such as drug delivery systems, tissue engineering, diagnostics, and the monitoring of small analytes such as emerging pollutants, is a result of their versatility. The utilization of phage display technology to identified novel peptides become an alternative way nowadays. The identified peptides with proper features very often contain specific motifs that are well known in biotechnology field.

The incorporation of fluorophores and the precise control over peptide sequences have been made possible by advanced techniques in peptide synthesis, such as solid-phase peptide synthesis. These developments have the potential to further improve the properties of peptide-based materials. It is possible to develop materials with specific chemical and biological properties that are specifically tailored to specific applications by customizing the sequence and structure of peptides.

In summary, we developed a highly selective and sensitive fluorescent chemosensor for Cu^2+^ detection based on a peptide from a phage display library that carries the N-terminal Xxx-His motif. This recognition could lead to more targeted approaches in designing peptides with an enhanced binding affinity for copper(II) ions.

## 5. Patents

The results of this work were included under two patent application numbers: PL449080 and PL449085.

## Data Availability

The datasets used and analyzed in the current study are available from the corresponding author upon reasonable request.
